# Dasatinib/HP-β-CD Inclusion Complex Based Aqueous Formulation as a Promising Tool for the Treatment of Paediatric Neuromuscular Disorders

**DOI:** 10.3390/ijms20030591

**Published:** 2019-01-30

**Authors:** Annalisa Cutrignelli, Francesca Sanarica, Antonio Lopalco, Angela Lopedota, Valentino Laquintana, Massimo Franco, Brigida Boccanegra, Paola Mantuano, Annamaria De Luca, Nunzio Denora

**Affiliations:** 1 Department of Pharmacy-Drug Sciences, University of Bari “Aldo Moro”, 70125 Bari, Italy; francesca.sanarica88@gmail.com (F.S.); antonio.lopalco@uniba.it (A.L.); angelaassunta.lopedota@uniba.it (A.L.); valentino.laquintana@uniba.it (V.L.); massimo.franco@uniba.it (M.F.); brigida.boccanegra@uniba.it (B.B.); paola.mantuano@uniba.it (P.M.); annamaria.deluca@uniba.it (A.D.L.); 2 Unity of Pharmacology, Department of Pharmacy-Drug Sciences, University of Bari “Aldo Moro”, 70125 Bari, Italy

**Keywords:** dasatinib, Duchenne muscular dystrophy, cyclodextrin inclusion complex, phase solubility studies, paediatric age, liquid formulation

## Abstract

New scientific findings have recently shown that dasatinib (DAS), the first-choice oral drug in the treatment of chronic myeloid leukemia (CML) for adult patients who are resistant or intolerant to imatinib, is also potentially useful in the paediatric age. Moreover, recent preclinical evidences suggest that this drug could be useful for the treatment of Duchenne muscular dystrophy, since it targets cSrc tyrosin kinase. Based on these considerations, the purpose of this work was to use the strategy of complexation with hydroxypropyl-β-cyclodextrin (HP-β-CD) in order to obtain an aqueous preparation of DAS, which is characterized by a low water solubility (6.49 × 10^−4^ mg/mL). Complexation studies demonstrated that HP-β-CD is able to form a stable host-guest inclusion complex with DAS with a 1:1 apparent formation constant of 922.13 M^−1^, as also demonstrated by the Job’s plot, with an increase in DAS aqueous solubility of about 21 times in the presence of 6% *w*/*v* of HP-β-CD (0.014 mg/mL). The inclusion complex has been prepared in the solid state by lyophilization and characterized by Fourier Transform Infrared (FT-IR), Nuclear Magnetic Resonance (NMR), Differential Scanning Calorimetry (DSC) techniques, and its dissolution profile was studied at different pH values. Moreover, in view of potential use of DAS for Duchenne muscular dystrophy, the cytotoxic effect of the inclusion complex has been assessed on C2C12 cells, a murine muscle satellite cell line. In parallel, a one-week oral treatment was performed in wild type C57Bl/6J mice to test both palatability and the exposure levels of the new oral formulation of the compound. In conclusion, this new inclusion complex could allow the development of a liquid and solvent free formulation to be administered both orally and parenterally, especially in the case of an administration in paediatric age.

## 1. Introduction

**Figure 1:**
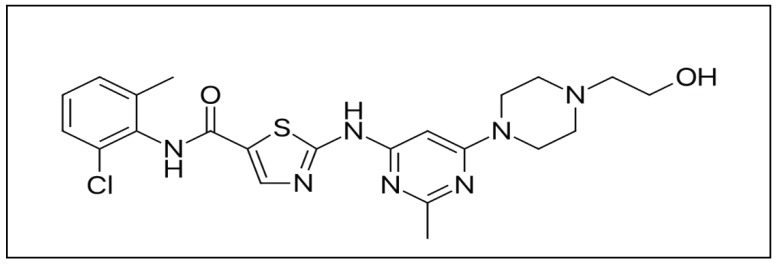
Chemical structure of dasatinib (DAS).

The drug dasatinib (DAS), whose chemical name is N-(2-chloro-6-methylphenyl)-2-[[6-[4-(2-hydroxyethyl)piperazin-1-yl]-2-methylpyrimidin-4-yl]amino]-1,3-thiazole-5-carboxamide (IUPAC) (Figure 1) is a double inhibitor of kinase proteins, including proto-oncogene tyrosine-protein Src (Src-TK) family kinases [1]. 

DAS is the first-choice oral drug in the treatment of chronic myeloid leukemia (CML) for those patients who are resistant or intolerant to imatinib. In fact, CML is a myeloprolifelative disorder that is caused by the BCR-ABL oncogene and DAS is a potent inhibitor of imatinib-resistant BCR-ABL mutants [2,3]. 

Until now, DAS was used exclusively for the treatment of adult patients, but new scientific findings have shown its potential in the treatment of CML in paediatric age, where its pharmacokinetic parameters, in particular, absorption and elimination time, were comparable with those in adult, with the same safety and efficacy profiles [4,5]. However, in these clinical trials, the drug was administered to children in the form of tablets or crushed tablets dispersed in fruit juice. In fact, DAS, formulated as monohydrate and marketed under the name of Sprycel® by Bristol Meyer Squibb, is presented in the form of coated tablets with a dosage ranging from 20 to 140 mg of the active ingredient. No liquid formulation is available on the market, and this may be a problem for paediatric patients who may not be able to swallow the tablets.

Moreover, recently, a study was published showing that DAS may be applied in the treatment of Duchenne muscular dystrophy (DMD), a genetic muscle-wasting disorder, whose symptoms occur around the age of four years in boys and get worse quickly. DMD is characterized by a progressive muscle degeneration and weakness and it is caused by the absence of the subsarcolemmal protein *dystrophin*. Dystrophin preserves sarcolemmal integrity by linking the cytoskeleton to the extracellular matrix via the interaction with the dystrophin-glycoprotein complex (DGC) and allowing for proper force transmission from contractile apparatus to extracellular matrix [6]. Thus, the primary structural defect causes an aberrant transmission of mechanical stimulus across the myofibers, leading to progressive muscle weakness and degeneration [7]. Similar defects occur in animals, such as the widely used C57Bl/10ScSn-Dmd*^mdx^*/J (*mdx*) mouse model [7,8]. Recent studies in *mdx* mouse model have highlighted that, in dystrophin deficient muscles, Src-TK is both overactivated and overexpressed, due to the excessive ROS production, and contribute also to NOX activation, in an auto-reinforcing loop [9], then playing a key role in DMD pathogenesis. In addition, Src-TK is involved in phosphorylation and degradation of β-dystroglycan (β-DG), a member of DGC, contributing to the loss of this complex in dystrophic myofibers. Thus, either the pharmacological inhibition of Src-TK seems a feasible strategy to ameliorate the pathology [10,11]. Src-TK inhibitors are already clinically available as antitumor drugs, and DAS belongs to this class of drugs.

Based on what has just been outlined, it is evident that it would be useful to develop a new formulation of DAS, which is different from the one currently in use, possibly liquid, so that it could be readily used in paediatric patients either by oral or parenteral route [12]. 

Therefore, the purpose of the following work was to prepare an aqueous formulation of this drug, evaluating the possibility of using an inclusion complex with cyclodextrins (CDs), as it is a molecule that is characterized by a low water solubility [13]. Cyclodextrins, cyclic oligosaccharides consisting of glucose units joined by α 1,4-glycosidic bond have been widely used to improve the solubility and stability in water of different molecules due to their ability to form host-guest inclusion complexes [14,15,16,17,18,19,20]. Thus, in this work, we present an inclusion complex of DAS with the hydroxy-β-cyclodextrin (HP-β-CD), a semisynthetic cyclodextrin that is approved by FDA also for the parenteral administration. The DAS/HP-β-CD inclusion complex was first studied in solution by building the phase solubility diagram according to Higuchi-Connors [21] and a two-dimensional-NMR (2D-NMR) Heteronuclear Multiple Bond Correlation HMBC evaluation was carried out in order to investigate the portion of the molecule actually contained in the HP-β-CD cavity. Subsequently, this complex was prepared in solid state by lyophilization and characterized by Fourier Transform Infrared (FT-IR), Differential Scanning Calorimetry (DSC), evaluation of the incorporation degree, and study of dissolution profiles at different pH values. Finally, in view of potential use of DAS for DMD, we first assessed its cytotoxic action on C2C12 cells, a muscle satellite cell line; secondly, we conducted an in vivo study in wild type C57Bl/6J (WT) mice by administering the inclusion complex in drinking water for one week to test both palatability and the exposure levels of the complex. 

## 2. Results and Discussion

### 2.1. Evaluation of the Inclusion Complex in Solution

First of all, the solubility of DAS was determined at 25 °C both in ultra-pure water and in buffered aqueous solutions at pH 1.2 (HCl 0.05 M, for oral administration) and at pH 7.4 (phosphate buffer 0.05 M, for parenteral administration). The results are shown in Table 1. DAS is a strong base with a pK_a_ value of 10.28 [11], so it is more soluble in acid environments where the protonation of the NH groups occurs.

**Figure 2:**
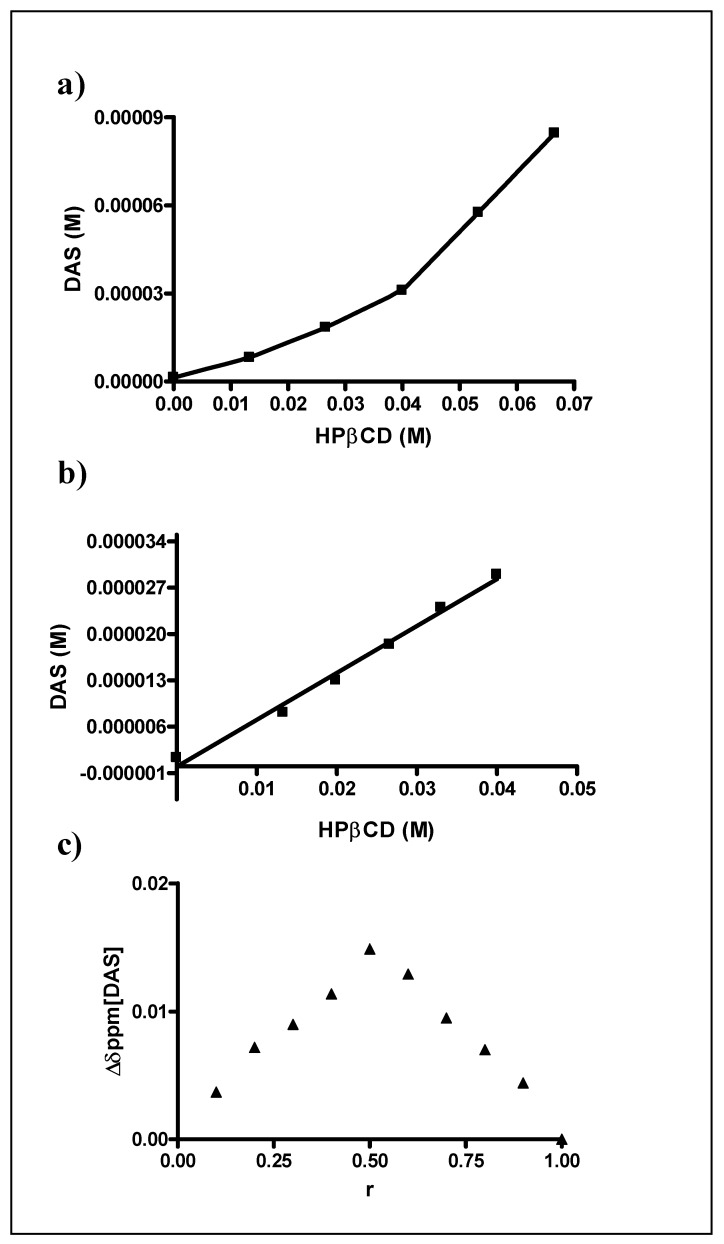
Phase solubility and Job’s plot diagrams of DAS and hydroxypropyl-β-cyclodextrin (HP-β-CD) in water at 25 °C. (**a**) Phase solubility diagram in the HP-β-CD concentration range 0–10%; (**b**) Phase solubility diagram in the HP-β-CD concentration range 0–6%; (**c**) Job’s plot diagram.

The lowest solubility value was recorded in ultrapure water with a pH value of about 6.0, so the phase solubility diagram relating to the complexation of DAS with HP-β-CD has been studied at 25 °C in water. According to the Higuchi and Connors classification [20], it shows an A_P_-type profile, as reported in Figure 2a, and this result clearly show that DAS solubility in water is linearly influenced by the presence of HP-β-CD until a percentage of cyclodextrin equal to about 6%, with the formation of an inclusion complex with 1:1 host:guest stoichiometry, while in the presence of major cyclodextrin percentages the formation of complexes with different stoichiometry occurs. From the analysis of the first linear portion of the Higuchi-Connors diagram (Figure 2b), it is possible to obtain the complexation constant for the complex with 1: 1 host:guest stoichiometry and it was found to be equal to 922.13 M^−1^, with an increase of about 21 times of the DAS solubility in the presence of 6% *w*/*v* of cyclodextrin (0.014 mg/mL, 2.9 × 10^−5^ M) as compared to the solubility value of the drug in the absence of the complexant, which results to be 6.49 × 10^−4^ mg/mL (1.33 × 10^−6^ M).

In order to determine the exact stoichiometric ratio between DAS and HP-β-CD in the formation of the inclusion complex, the Job’s plot (Figure 2C) was constructed, as described in the experimental section. In detail, this study was conducted via ^1^H-NMR observing the variation of chemical shifts of methyl hydrogens (CH_3_) on the pyrimidine ring of DAS. 

As shown in the graph, a highly symmetrical trend with a maximum value being recorded at *r* = 0.5 is observed, and this finding highlights the formation of a 1:1 inclusion complex. This result is quite in agreement with the phase solubility diagram because for the construction of Job’s plot very low concentration of HP-β-CD are used and at low concentration of cyclodextrin the formation of an inclusion complex with a 1:1 host:guest stoichiometry occurs. This behavior has already been widely described in the literature [15,22]. It is in fact known that the balance of complexation between drug and cyclodextrin is strongly influenced by the concentrations in solution of the two components and that in the presence of high concentration of cyclodextrin different solubilization phenomena take place that modify the stoichiometry of the inclusion complex, leading to higher-order complexes.

Furthermore, the construction of the Job’s diagram has been carried out on the basis of the displacement, in terms of chemical shift, of the methyl group protons on the DAS pyrimidine ring. It would therefore seem that this methyl group is directly involved in the formation of the inclusion complex with the cavity of the cyclodextrin and in order to obtain more information about the interactions of the drug with the cyclodextrin in solution, a ^1^H- and 2D-NMR (HMBC) study was conducted, keeping the DAS concentration constant and varying the molar ratio DAS: HP-β-CD.

**Figure 3:**
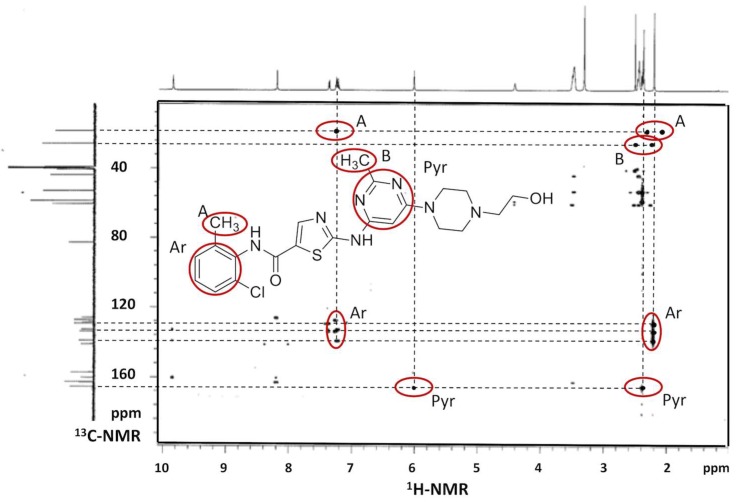
2D ^1^H- ^13^C-NMR spectrum of DAS in DMSO-d_6_. The cross-peaks displayed by HMBC were used to identify the structure of the drug, including the correlation of the δ of hydrogens and carbons separated from each other with two and three chemical bond.

**Figure 4:**
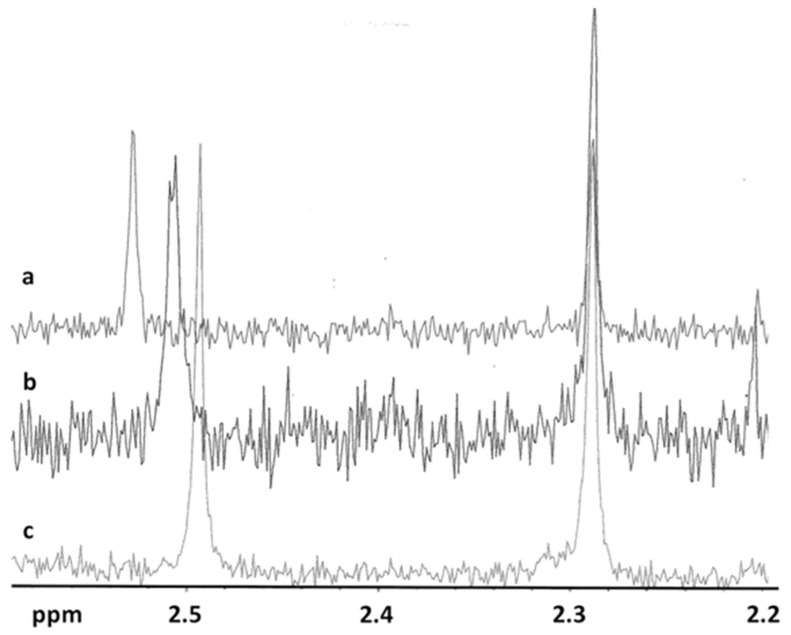
^1^H-NMR spectra of methyl resonances of CH_3_ on pyrimidine (δ ~2.5) and benzene (δ ~2.3) rings in the presence of cyclodextrin at different DAS:HP-β-CD molar ratios. (**a**) DAS:HP-β-CD molar ratios 1:0; (**b**) DAS:HP-β-CD molar ratios 1:1; and (**c**) DAS:HP-β-CD molar ratios 1:10.

Figure 3 shows the 2D ^1^H- ^13^C-NMR spectrum of DAS in DMSO-d_6_, which was used to make the correct assignment of DAS protons while in Figure 4a–c are reported the ^1^H-NMR spectra of methyl resonances of CH_3_ on pyrimidine and benzene rings at different DAS: HP-β-CD molar ratios.

The relative positions of the peaks were in agreement with the assignment. ^1^H- and 2D-NMR (HMBC) investigations confirmed the structure of the molecule and elucidated the interactions with the cyclodextrin in solution. Our results give a direct evidence of the formation of an inclusion complex between the drug and the cyclodextrin. In Table 2, we reported the variation of chemical shifts of methyl hydrogens (CH_3_) on the pyrimidine ring of DAS in the presence of different concentrations of cyclodextrin (i.e., molar ratio drug:cyclodextrin 1:1, 1:2, and 1:3). As one can see from Table 2, the chemical shifts of this CH_3_ are affected during complexation, showing changes in the ppm values. In particular, as shown in Figure 4a–c increasing the concentration of cyclodextrin in solution, we observed that the chemical shift of the CH_3_ hydrogens on the pyrimidine ring shifted downfield (higher ppm). These findings suggest that the methyl hydrogens on the pyrimidine ring were directly involved in the complexation with cyclodextrin. In detail, the hydrogen nuclei of the drug included in the cyclodextrin cavity established hydrophobic interactions with cyclodextrin hydrogens, resulting in a their deshielding. No significant variation of the chemical shifts of the methyl hdrogens on the aromatic ring benzene was observed. This would suggest that this portion of the molecule is not interested in the complex formation with cyclodextrin.

### 2.2. Characterization of the Inclusion Complex in the Solid State

**Figure 5:**
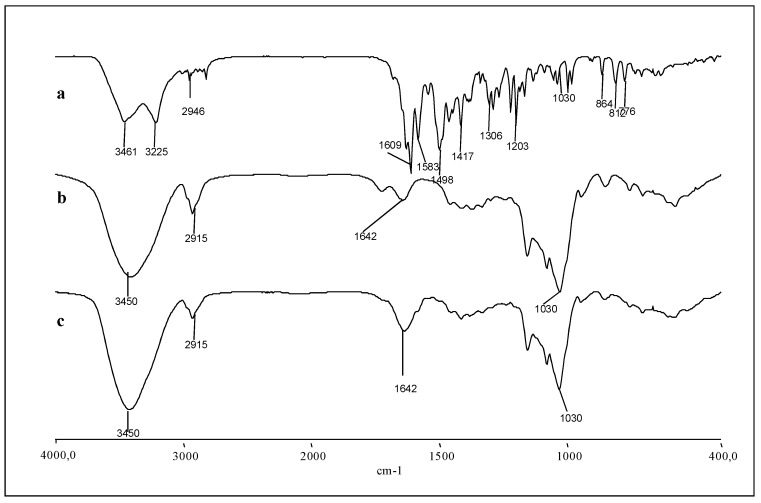
Fourier Transform Infrared (FT-IR) spectra (**a**) DAS, (**b**) HP-β-CD, and (**c**) DAS/HP-β-CD complex.

In order to exploit the complexation with HP-β-CD in the preparation of a powder formulation of the drug that instantly dissolves when placed in water to be administered orally or parenterally, the solid-state complex was prepared by lyophilization. The freeze-dried complex was characterized by the assessment of the degree of incorporation, expressed as g of DAS per 100 g of product and it was found to be 4.23 ± 0.42 g of DAS per 100 g of lyophilized powder. This solid inclusion complex has been characterized by FT-IR, DSC, and dissolution profile. Figure 5 shows the IR spectra of DAS, HP-β-CD, and HP-β-CD solid inclusion complex. 

The IR spectrum of DAS shows an absorption band at 1609 cm^−1^ due to stretching of the carbonyl group in the amidic bound, and two absorption bands at 2945 and 2930 cm^−1^ due to C-H stretching of methylenic and alchilic groups. In addition, the bands at 1583, 1498, and 1417 cm^−1^ corresponding to the C-C strain of the aromatic ring, and the bands at 3461 and 3225 cm^−1^, corresponding to the stretching of the N-H and O-H are highlighted, respectively.

**Figure 6:**
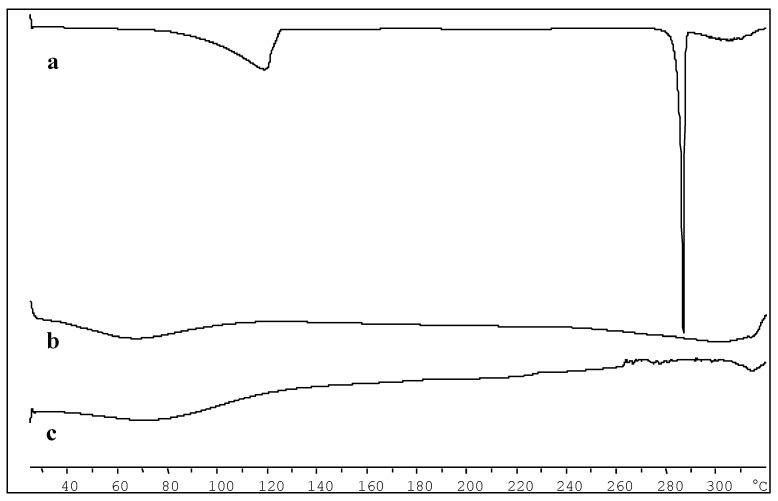
DSC thermograms: (**a**) DAS, (**b**) HP-β-CD, and (**c**) DAS/HP-β-CD complex.

The spectra of CDs inclusion complex appear to be very similar to those of cyclodextrin, since the cyclodextrins exhibit a high number of polar groups (OH, CO) that give rise to very broad absorption bands, which in some regions often overlap with those of DAS, also because a large excess of cyclodextrin is present in the complexes. This finding is confirmed by the DSC study reported in Figure 6.

In the DAS thermogram (Figure 6a), it is evident the crystalline nature and the high degree of purity of this compound that shows an endothermic spike at 285 °C, according with data reported in literature [11].

The HP-β-CD thermogram (Figure 6b) highlights the amorphous nature of the same, which does not exhibit an endothermic melting peak but only a sloping peak between 80 and 100 °C due to the loss of the water present in the sample. The thermogram of the DAS/HP-β-CD complex (Figure 6c) has a trend that is comparable to that of cyclodextrin alone and this indicates the drug’s inclusion within the cavity of the complexing agent with its amorphization.

### 2.3. Dissolution Studies

**Figure 7:**
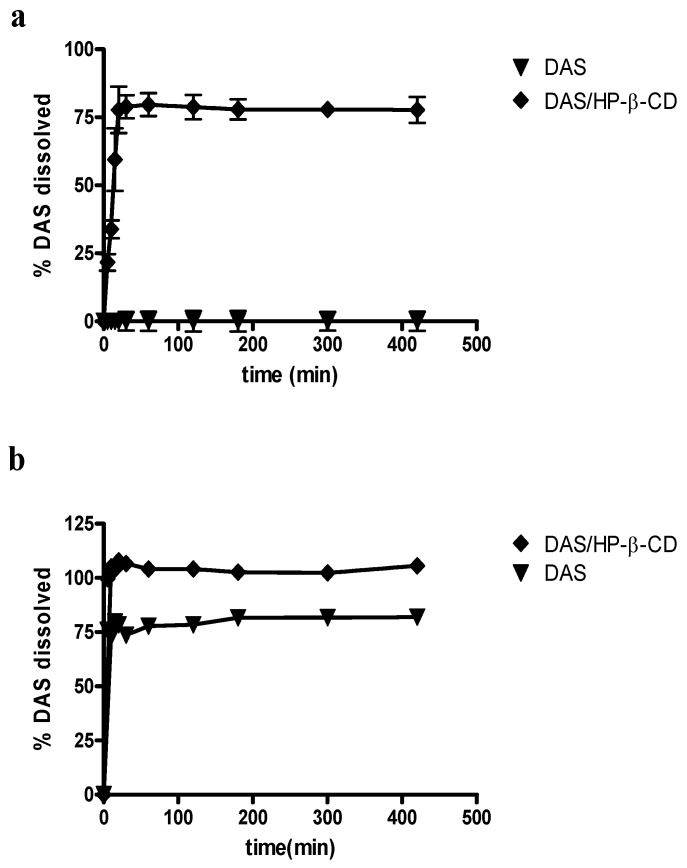
Dissolution profiles at 37 °C: (**a**) pH 7.4 and (**b**) pH 1.2 of DAS alone (▼) and DAS/HP-β-CD complex (♦). All values are mean ± SD, *n* = 3.

Moreover, dissolution studies have been performed at 37 °C in two different media: phosphate buffer 0.05M pH = 7.4 and HCl 0.05M pH = 1.2. Figure 7 shows obtained dissolution profiles. 

It is clear that the DAS/HP-β-CD lyophilized complex exhibits a better dissolution profile than the drug alone. In particular, this is especially evident at pH 7.4 where it was not possible to obtain the dissolution profile of DAS alone due to its very low solubility at this pH value, which prevents the quantitative determination of the drug via HPLC in the dissolution medium. The hydrophobic nature of the drug prevented its contact with the dissolution medium, causing it to float on the surface and hindering its dissolution. Instead, in the same dissolution medium the presence of HP-β-CD allows for the achievement of a quantity of dissolved drug equal to about 77% after 420 min. At pH 1.2, DAS appears to be more soluble, as demonstrated by the previously described solubility analysis. In this case, hence, it was possible to obtain the solubility profile of the drug alone at this pH value and it is evident that after approximately 420 min the quantity of drug dissolved is approximately 81%, as compared to the 100% that is reached from the complex with the HP-β-CD.

Therefore, the complexation with the selected cyclodextrin certainly represents an effective strategy for improving the solubility characteristics and the dissolution profile of DAS, also allowing an improvement of these characteristics with respect to those of monohydrate and polymorphic forms that are patented [23,24], and enabling it to be administered parenterally, in addition to oral administration, which is currently the only possible DAS route of administration.

### 2.4. Cytotoxicity Studies

**Figure 8:**
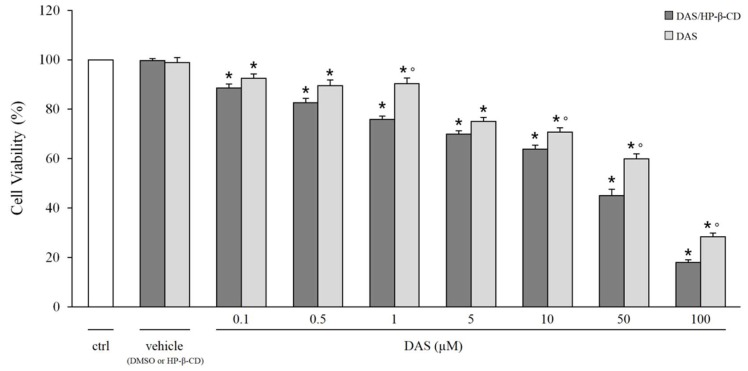
Effect of DAS on cell viability. The figure shows the cytotoxic effect on cell viability of increasing concentration of DAS (0.1–100 μM) alone or complexed with HP-β-CD. The results are expressed as the percentage of the control (ctrl) and presented as the mean ± S.E.M. Each data is from 24–48 replicates (wells) and 6–9 different culture dishes. The statistical significance between groups was evaluated by Student’s *t*-test, as follows: significantly different with respect to * the control value (0.001 < *p* < 0.05); ° DAS/HP-β-CD at the same concentration (0.001 < *p* < 0.05).

The cell viability study was performed to compare the cytotoxicity, and then the pharmacological activity, of the DAS/HP-β-CD inclusion complex with that of the free drug both solubilized in DMEM. In view of potential use for DMD, this effect has been assessed on C2C12 myoblasts. For DAS alone, due to its very low water solubility, a DMSO solution was prepared and this solution was subsequently diluted in DMEM so that the final DMSO concentration in each well was less than 0.15% in order to ensure cellular vitality. The two vehicles were also tested, i.e., HP-β-CD and DMSO, both diluted in DMEM and both at the highest concentration tested in the presence of DAS. The results that were obtained in terms of cellular viability are shown in Figure 8. 

It is clear that all of the compounds tested show, as expected, a cytotoxicity that is concentration dependent. In particular, the DAS/HP-β-CD complex has a relatively higher effect on cell viability than free DAS, with significantly different statistical results (0.001 < *p* < 0.005 and 0.025 < *p* <0.001). Furthermore, since both vehicles are not cytotoxic, because they guarantee 100% cellular viability, the cytotoxicity recorded in the test is attributable exclusively to the effect of the drug. The obtained result suggests that DAS complexation with HP-β-CD increases the cytotoxicity of the drug, and this effect is probably a consequence of the increased solubility of DAS in water-like phase. Anyway, it is important to underline that the concentration at which DAS exerted cytotoxic actions on C2C12 cells, both free than complexed with HP-β-CD, is higher that the IC50 values known to inhibit cancer cell growth, which are in the nM range. Therefore, this in vitro experiment underlines that DAS is relatively safe on satellite muscle precursors being cytotoxic only at high concentrations. In fact, the concentrations that are used in the cell viability test are above the therapeutic plasma levels of DAS, which range in the low µM values.

### 2.5. Pharmacokinetic Results

**Figure 9:**
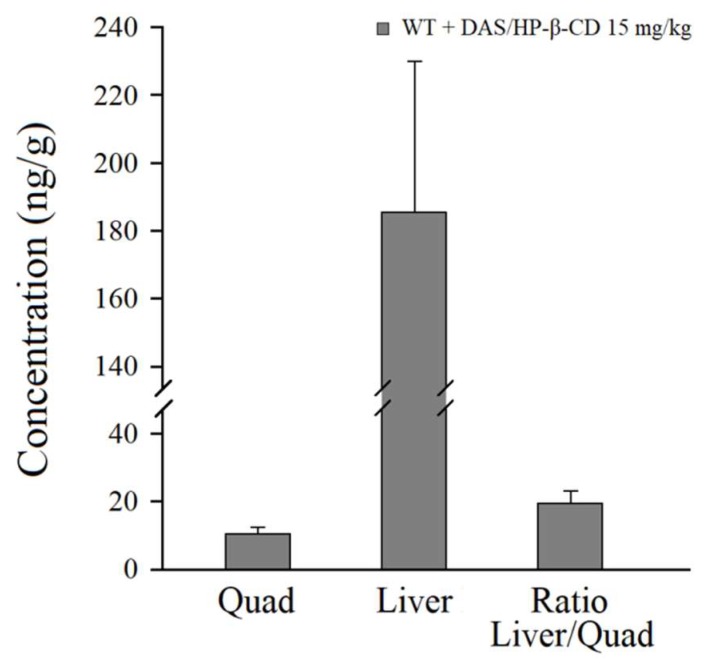
Pharmacokinetic analysis in quadriceps and livers of DAS/HP-β-CD inclusion complex administered at 15 mg/kg in drinking water for 1 week. All values are mean ± S.E.M. from 7–8 mice for each group. No significant difference was found by Student *t*-test analysis.

HPLC analyses were carried out to evaluate the DAS traceability in main target tissues (quadriceps and liver) of treated mice. Appreciable drugs’ levels were found in quadriceps and livers of treated animals (Figure 9). These results are in line with the finding that DAS is rapidly distributes in tissues [25]. Also, the level reached in skeletal muscle allows for predicting sufficient exposure for the action of DAS to take place, considering that the inhibition of Src-TK occurs in the nanomolar range [26].

## 3. Materials and Methods

### 3.1. Materials

DAS (MW = 488 g/mol), was purchased from Sigma Aldrich (Milan, Italy). HP-β-CD (hydroxypropil-β-cyclodextrin, MW = 1396 Dalton, substitution degree 0.65) was kindly provided by Roquette (FR). HCl and phosphate salts for the preparation of buffers were purchased from Fluka (Sigma Aldrich, Milan, Italy). Bidistilled water was bought from Carlo Erba (Milan, Italy). The cell counting Kit-8 (CCK-8) used for cytotoxicity studies was purchased from Sigma Aldrich (Milan, Italy). All other products and reagents used in this work were of analytical grade.

### 3.2. Quantitative Analysis of DAS

The quantitative analysis of DAS was performed by High-performance liquid chromatography (HPLC). In detail, a HPLC station composed of a Agilent 1260 LCVL quaternary pump, a variable wavelength UV-visible detector and a fixed 20 μL loop manual injector was used. The analytical data were processed with the Agilent OpenLab LC software. For the analysis, a C_18_ Zorbax SB – Aq (4.6 × 150 mm) column was eluted in isocratic mode with a methanol/ammonium acetate pH = 3 60/40 *v*/*v* mixture, continuously monitoring the eluent at 280 nm. In these conditions, the retention time of the drug was about 2.8 min.

Standard calibration curves were prepared at a wavelength of 280 nm using the same analysis conditions and they resulted in a linear plot (*r*^2^ = 0.999) in the range of tested concentrations (from 2.17 × 10^−4^ M and 6.78 × 10^−6^ M).

### 3.3. Solubility and Phase-Solubility Studies

The phase solubility study was conducted in accordance with Higuchi and Connors [21]. In detail, DAS was added in excess to an aqueous solution containing HP-β-CD in the appropriate concentration (0–10% *w*/*v*) until saturation and the suspensions thus obtained were placed in 4 mL vials with screw cap to avoid changes that are caused by evaporation. The obtained mixtures were vortexed for about 5 min and then placed in a thermostat bath at 25 °C for three days.

Subsequently, an aliquot of the aqueous phase of each mixture was transferred into a 5 mL glass syringe and filtered through a 0.22 μm cellulose acetate membrane filter (Millipore®, Milan, Italy).

The obtained filtrate was suitably diluted and subjected to subsequent HPLC analysis for the quantification of the drug. All of the determinations were conducted in triplicate.

The obtained data were used to determine the apparent stability 1:1 constant (K_1:1_) of the DAS/HP-β-CD inclusion complex, using the slope of the phase solubility diagrams straight line, as reported by Higuchi and Connors in the following equation:

(1) $$K_{1:1}=\frac{slope}{S_{0}(1-slope)}$$

where *S*_0_ represents DAS solubility in absence of cyclodextrin determined in the same way.

### 3.4. Preparation of Solid DAS /HP-β-CD Inclusion Complex

The DAS/HP-β-CD inclusion complex was prepared in the solid state by freeze drying [18]. The lyophilized product was prepared by adding DAS and HP-β-CD in water in equimolar amounts. The obtained suspension was vigorously vortexed for about five minutes, left under stirring for two days, filtered through 0.22 μm cellulose acetate filters (Millipore), then frozen, and lyophilized (Lio 5P, Milan, Italy). The obtained product was characterized by DSC and FT-IR.

### 3.5. Determination of DAS Incorporation Degree in the Solid Cyclodextrin Inclusion Complex

The amount of DAS that is present in the DAS/HP-β-CD solid complex was determined by solubilizing about 5 mg of sample in 5 mL of deionized water. Samples were injected in HPLC after filtration with 0.22 μm cellulose acetate filters (Millipore®). The incorporation degree of DAS into the inclusion complex was determined from the peak areas obtained and expressed as g of DAS per 100 g of complex.

### 3.6. Job’s Plot Method

The stoichiometry of the inclusion complex DAS/HP-β-CD in aqueous solution was determined by the continuous variation method or Job’s method [15]. Briefly, equimolar (1.02 × 10^−3^ M) CD_3_OD/D_2_O (50/50, *v*/*v*) solutions of DAS and HP-β-CD were mixed to a fixed volume by varying the molar ratio from 0 to 1, keeping the total molar concentration of the species constant. After stirring for 1 h, for each solution the ^1^H-NMR spectra were registered and the chemical shifts of the host’s protons were calculated and expressed as ppm. The Δchemical shift was determined as the difference between chemical shifts with and without HP-β-CD. Subsequently, Δppm × [DAS] was plotted versus *r*, where:

(2) $$r=\frac{\left[DAS\right]}{\left[DAS]+[HP-\beta -CD\right]}$$

### 3.7. ^1^H-NMR and Heteronuclear Multiple Bond Correlation (HMBC) Spectroscopic Studies

^1^H nuclear magnetic resonance (^1^H-NMR) spectra were recorded using a NMR Agilent Technologies 500/54 Premium Shielded instrument and ^1^H chemical shifts were referred to DHO as internal standard. For the Heteronuclear Multiple Bond Correlation (HMBC) experiment, an Agilent 500 mHz spectrometer was used. The concentration of the drug was 10 mg/mL in a 5-mm NMR tube. Sample temperature was set to 25 °C. The following parameters were used for 2D ^1^H- ^13^C- heteronuclear multiple bond correlation (HMBC) experiment: number of scans, 2, number of complex data points (experiments) in F1, 128; number of complex data points in F2, 2048; sweep width in F1 and F2, 222 and 13ppm, respectively; spectrometer offset for ^1^H and ^13^C, 6 and 100 ppm, respectively; interscan delay, 1.5 s. Data were processed with the software Topspin. For 2D, the spectrum was zero filled to 512 data apodization function in both dimensions prior to Fourier transform and phase correction. Chemical shifts were expressed in parts per million (ppm) with respect to the DMSO-d_6_ signal for carbon and H_2_O_2_ for proton [27,28,29,30,31].

### 3.8. Fourier Transform Infrared (FT-IR) Spectroscopy

The FT-IR spectra of DAS, HP-β-CD, and DAS/HP-β-CD solid complex were recorded with a Perkin-Elmer 1600 FTIR spectrophotometer dispersing each sample in KBr for spectroscopy (2 mg of sample in 200 mg of KBr) [32]. The scan range used was 400–4000 cm^−1^, with a resolution of 1 cm^−1^. The instrument was periodically calibrated.

### 3.9. Differential Scanning Calorimetry (DSC) Analysis

The thermal analysis of DAS and DAS/HP-β-CD solid complex were performed using a Mettler Toledo DSC 822e Star^e^ 202 system (Mettler Toledo, Switzerland) equipped with a thermal analysis automatic program, as described in a previous work [33]. The instrumentation was calibrated periodically, using indium as reference.

### 3.10. Dissolution Studies

Dissolution experiments were performed at 37 °C using a BIODIS USP III apparatus (Varian Inc., Cary North Carolina, CA, USA), equipped with a rod stirrer maintaining a rotational speed of 100 rpm during the test. Samples of DAS or DAS/HP-β-CD solid complex, equivalent to about 2 mg of DAS, were suspended in the dissolution medium (80 mL of 0.05 M phosphate buffer at pH 7.4 or HCl 0.05 M pH = 1.2). The volume of 80 mL was chosen taking into account the HPLC quantification limit for the determination of DAS.

At predetermined time intervals, 600 µL of suspension were collected and, in order to keep constant the initial volume, 600 μL of the same dissolution medium previously thermostated at the same temperature were added. Samples were subsequently filtered using a 0.22 µm membrane filter (Millipore® cellulose acetate), and the filtrates thus obtained were subjected to HPLC analysis after appropriate dilution. For quantitative analysis the calibration curve previously constructed was used and the dissolution profiles shown correspond to the average of three determinations.

### 3.11. Cytotoxicity Studies

C2C12 myocytes were cultured in DMEM that was supplemented with 10% fetal bovine serum, 1% penicillin, 1% streptomycin and 1% glutamine and were maintained at 37 °C in 5% CO_2_/95% air. Cell viability was evaluated by measuring the succinic dehydrogenases activity in the cell suspension using the cell counting Kit-8 (CCK-8) (Sigma Aldrich), which utilizes a highly water-soluble tetrazolium salt and whose detection sensitivity is higher than other tetrazolium salts [34].

Cells were seeded in 96-well cultures at a density of approximately 4.5 × 10^3^ cells per well and then cultured for 16 h. Afterwards, the cells were treated for 5 h with free DAS (in DMSO <0.15% in order to ensure cellular vitality) or complexed with HP-β-CD, but at the same concentration calculated on the basis of the incorporation degree, both being dissolved in DMEM. Following exposure, 10 μL of CCK-8 were added into each well and then the plate was incubated for additional two hours. The absorbance at 450 nm was measured using a spectrophotometer (microplate reader Victor V31420–40; PerkinElmer, Wellesley, Massachusetts). Cell viability (%) is expressed according to the following formula:

cell viability (%) = [(test value − blank)/(control value − blank) × 100](3)

where the blank value represents that of a cell-free wells; the control value represents that of wells of cells do not treated with DAS and the test value represents that of wells of cells treated with DAS. The results are expressed as the percentage of the control and presented as the mean ± SD. Each data is from 24–48 replicates (wells) and 6–9 different culture dishes.

### 3.12. In Vivo Study

A total of 10 sedentary WT male mice C57Bl/6J (Charles River, Italy for Jackson Laboratories), homogeneous for age and body weight (BW) were divided into 2 groups as follows: 4 WT mice vehicle-treated (HP-β-CD 10%) and 6 WT mice treated with DAS/HP-β-CD inclusion complex at the dose of 15 mg/Kg. Drug and vehicle were administered in drinking water for 1 week. The dose was chosen based on data in literature, in fact, the human dose commonly administered in clinical practice, converted in the appropriate animal equivalent, resulted to be approximately 20 mg/kg per day [35]. Care in animal handling and environment conditions was used to avoid any animal discomfort and stress during the study period. Food intake was monitored, and composition was maintained constant [36]. No abnormal gross findings in animal well-being and no animal deaths were observed during the study period.

### 3.13. Ex vivo Study: Pharmacokinetic Analysis

The pharmacokinetic (PK) analysis were commissioned to the CRO “XenoGesis Ltd.—Preclinical DMPK & Bioanalysis services, Nottingham, UK. In detail, analysis was performed in quadriceps (Quad) and livers of treated animals. Tissues were individually weighed into a "FastPrep" tube and PBS was added (3:1 ratio). Each tube was placed in the fast prep homogenizer on a predetermined 1min cycle to ensure complete homogenization. 40 µL of each homogenate was aliquoted to a fresh tube and 50 µL of MeOH plus 150 µL of Methanol-containing Internal standard (25 ng/mL Imipramine HCl) was added. Each sample was mixed on a Bioshake for 1 min and then transferred to the freezer at −20 °C for at least two hours prior centrifugation at 2500 × *g* for 20 min. The supernatants were then transferred to a 96-well plate for sampling by the LC-MS/MS. A Thermo TSQ Quantiva with Thermo Vanquish UHPLC system was used (Thermo Fisher Scientific Inc, Milan, Italy). Separation was achieved on a ACE-AR C18 (50 × 2.1 mm, 1.7 µm) column, with MilliQwater 0.1% formic acid (solvent A) and methanl-0.1% formic acid (solvent B) at 65 °C and at a flow rate of 0.8 mL/min. Positive ion spray voltage and vaporizer temperature were set at 3500 V and 450 °C, respectively, while the ion transfer tube temperature was set at 365 °C. Finally, sheath gas and auxiliary gas pressures were fixed at 54 and 17 bar, respectively. Detection was performed using a multiple reaction monitoring (MRM) via a positive ESI source spray voltage. Quantitative analysis was conducted by MRM at 232.06 to 401.11 *m*/*z* for DAS inclusion complex and at 86.10 to 193.04 *m*/*z* for the internal standard Imipramine. Mass transitions were combined for each compound to maximize sensitivity.

### 3.14. Statistic

In the elaboration of results in the cytotoxicity studies, the statistical significance between groups was evaluated by Student’s *t*-test, as follows: * significantly different with respect to control value (0.001 < *p* < 0.005); significantly different with respect to DAS complexed with HPβCD (0.025 < *p* < 0.001).

## 4. Conclusions

From the results that were obtained in this study, it is possible to state that the complexation of DAS with HP-β-CD is successful both in solution and in the solid state. In particular, the presence of the cyclodextrin allows for obtaining an increase in the drug water solubility and a favorable dissolution profile especially at pH 7.4 as compared to the non-complexed drug Moreover, cytotoxicity studies highlight that DAS complexation with HP-β-CD increases the cytotoxicity of the drug and PK results of a one-week pilot study with DAS/HP-β-CD inclusion complex in WT mice provided the basis for further long-term in vivo treatment with this new oral formulation of DAS in treadmill-exercised *mdx* mice.

In conclusion, this new inclusion complex could allow the development of a liquid formulation to be administered orally, which could be a valid alternative to the one currently present on the market that is solid, especially in the case of an administration in paediatric age. Moreover, considering that HP-β-CD is FDA approved for parenteral formulations, the DAS/HP-β-CD inclusion complex could also be an interesting tool for the administration of DAS by this route.

## Figures and Tables

**Table 1:** DAS solubility at 25 °C in presence of different environments.

Environment	DAS Solubility (mg/mL)	DAS Solubility (M)
**HCl 0.05 M** pH 1.2	4.31 × 10^−^^2^	8.84 × 10^−^^5^
**Phosphate Buffer 0.05 M** pH 7.4	7.65 × 10^−^^4^	1.56 × 10^−^^6^
**Water**	6.49 × 10^−^^4^	1.33 × 10^−^^6^

**Table 2:** Shifts of CH_3_ hydrogens in the presence of cyclodextrin at different DAS: HP-β-CD molar ratios.

Molar Ratio DAS: HP-β-CD	δ CH_3_ (Pyrimidine)	δ CH_3_ (Benzene)
1:0	2.498	2.292
1:1	2.513	2.293
1:10	2.534	2.294
